# RON Receptor Signaling and the Tumor Microenvironment

**DOI:** 10.3390/genes16040437

**Published:** 2025-04-06

**Authors:** Emily Wachter, Levi H. Fox, Zhixin Lu, Angelle D. Jones, Nicholas D. Casto, Susan E. Waltz

**Affiliations:** 1 Department of Cancer Biology, University of Cincinnati College of Medicine, Cincinnati, OH 45267-0521, USA; 2 Research Service, Cincinnati Veterans Affairs Hospital Medical Center, Cincinnati, OH 45220, USA

**Keywords:** RON, RTK, HGFL, immune microenvironment, tumor microenvironment, macrophages, mouse models, interferons, cancer, metastasis

## Abstract

The immune microenvironment plays a critical role in tumor growth and development. Immune activation within the tumor microenvironment is dynamic and can be modulated by tumor intrinsic and extrinsic signaling. The RON receptor tyrosine kinase is canonically associated with growth signaling and wound healing, and this receptor is frequently overexpressed in a variety of cancers. Epithelial cells, macrophages, dendritic cells, and fibroblasts express RON, presenting an important axis by which RON overexpressing tumors influence the tumor microenvironment. This review synthesizes the existing literature on the roles of tumor cell-intrinsic and -extrinsic RON signaling, highlighting areas of interest and gaps in knowledge that show potential for future studies.

## 1. Overview of RON Signaling

RON is a transmembrane receptor tyrosine kinase of the MET proto-oncogene family. The RON receptor is activated by binding of the ligand hepatocyte growth factor-like (HGFL) protein. HGFL binding induces RON dimerization and autophosphorylation, leading to downstream growth signaling via phosphorylation of Src Homology 2 domains [1]. RON signaling is associated with growth pathways in epithelial cells via downstream activation of AKT and MAPK signaling [1,2]. In many cancers, RON is overexpressed on epithelial cells, leading to unrestrained cell proliferation. RON signaling in macrophages is associated with wound healing in part due to the downstream increases in arginase which limits nitric oxide production to de-escalate the inflammatory response and to assist in wound repair [3].

## 2. The Tumor Microenvironment

The immune system plays a critical role in keeping an organism safe from external threats (such as bacteria, viruses, and parasites) and from internal threats such as cancer cells and compromised cells. Although belonging to separate branches of the immune system, lymphoid and myeloid cells work in tandem to defend, maintain, and repair the human body from both external and internal threats. In a typical immune response, foreign pathogens are recognized by their pathogen-associated molecular patterns, resulting in the recruitment of innate immune effectors such as tissue-resident macrophages, dendritic cells, neutrophils, and natural killer (NK) cells [4]. Dendritic cells at the site of inflammation can recruit the adaptive immune system (T cells and B cells) by engulfing a foreign pathogen, breaking it down into smaller fragments consisting of peptides or polysaccharides, and then presenting these peptides or polysaccharides as antigens on the cell surface for recognition by T-cells. This results in the activation and clonal expansion of T cells and B cells equipped to recognize the foreign pathogen. The macrophage-secreted chemoattractant gradient leads these T and B cells to the site of inflammation so that innate and adaptive effectors can work together to eliminate the infection. After the infection has been eliminated, the immune system has many processes in place to reduce inflammation and promote healing. Macrophages can secrete cytokines to promote healing of the local epithelial and stromal tissues [5]. Regulatory T cells and macrophages secrete cytokines to promote exhaustion, prevent proliferation, and induce apoptosis in other immune cells to shut down the immune response [5,6].

While the immune system typically prevents cancer from implanting in its early stages by recognizing and eliminating malignant cells, tumors have developed numerous methods of exploiting or evading the immune response [7]. Cancer cells can regulate the expression of certain cell surface proteins to prevent detection by cytotoxic T cells and natural killer cells capable of direct cell killing. Likewise, cancer cells may develop the ability to communicate with the immune system by releasing cytokines to alter activation, polarization, or infiltration of immune cells. This immunosuppressive tumor microenvironment makes further immune intervention far more difficult and inefficient [8]. Signaling from tumor cells also influences tumor-associated macrophages (TAMs). TAMs help support the tumor in numerous ways, whether by pro-growth signaling, anti-inflammatory cytokine signaling to prevent other immune cells from killing the tumor, secretion of matrix metalloproteinases (MMPs) to break down the extracellular matrix, secretion of VEGF for angiogenesis, promotion of epithelial-to-mesenchymal transition (EMT) in tumor cells, or even extravasation through blood vessels to help tumors metastasize [9,10]. One protein that enables crosstalk between tumor cells and the immune system is the RON receptor tyrosine kinase.

RON is overexpressed in a number of cancers and is naturally expressed in macrophages, specifically those residing in the local tissue [11]. Additionally, tissue-resident macrophages expressing RON have been shown to polarize into a pro-tumorigenic state [12,13,14,15]. Monocytes, the precursors to macrophages, do not express this receptor [16]. Since RON signaling is associated with growth and de-escalation of the immune response, the receptor plays a homeostatic role in macrophages in the promotion of wound healing [17]. However, overexpression of RON and its ligand by tumor cells can activate RON signaling in the tissue-resident macrophages to promote growth and inhibit inflammation. Thus, the activation of RON in both macrophages and cancerous cells has led to mounting evidence that such signaling promotes both tumor growth and metastasis through tumor microenvironment (TME) signaling [18,19,20].

## 3. Genetically Engineered Mouse Models (GEMM)

The use of mouse models to study human cancers has become common practice in many research settings. However, there are limitations to any model in studying tumor formation in human patients or the innate differences between human and mouse biology. In vivo modeling of cancer in mice remains crucial despite the limitations, as these models provide a more biologically relevant background for experimentation. Nowhere is the biological background more important than in microenvironment studies. The generation of murine models in immune-competent mice has allowed for a deeper understanding of the influence of RON signaling on the tumor microenvironment, tumor growth, metastasis, and tumor initiation. Below, we summarized the findings of a variety of genetically engineered mouse models to study RON receptor signaling and how genetic changes affect tumor characteristics and impact the tumor immune microenvironment (Table 1).

### 3.1. RON Knockout Mouse Models

To determine the functions of RON in vivo, a variety of genetically engineered mouse models have been developed. In one model, the deletion of the RON function was accomplished by targeting a large genomic region encompassing the RON extracellular and transmembrane domains (exons 1–14). This deletion resulted in early embryonic lethality before the peri-implantation period [21]. Conversely, RON knockout mice generated by targeted deletion of the first exon of RON develop normally with no obvious developmental defects [22]. However, these mice, referred to as STK (stem cell kinase receptor)/RON−/− mice, exhibit increased susceptibility to lipopolysaccharide-induced endotoxic shock and support a role for RON in dampening the inflammatory response. These studies, combined with information from further gene knockout studies, support the notion that RON loss in mice leads to viable offspring and that deletion of a large region surrounding this receptor may have resulted in early embryonic lethality.

An additional GEMM was also developed to examine the biological function of RON and include targeting the intracellular tyrosine kinase (TK) domain of RON. RON^F/F^ mice were generated by flanking the RON TK domain, exon 13 to exon 18, with loxP recombination sites [23]. Following Cre-mediated deletion, a global RON TK mouse was generated and termed RON TK−/− mice [23]. Adult RON TK−/− mice are phenotypically normal but have an altered ability to regulate nitric oxide levels and exhibit enhanced tissue damage following acute and cell-mediated inflammatory responses. Interestingly, in response to HGFL, peritoneal macrophages isolated from RON-replete mice undergo morphological shape changes indicative of activation, while macrophages from RON TK−/− mice do not respond to HGFL [23]. The first report describing the role of RON on breast tumorigenesis and metastasis in a murine model utilized the RON TK−/− mice crossed to mice expressing the polyomavirus middle T antigen (PyMT mice) specifically in the mammary epithelium [24]. RON loss in this model led to decreases in mammary tumor formation and metastasis and established RON signaling as a crucial regulator of mammary tumor formation, growth, and metastasis, as well as in the regulation of angiogenesis. Similar to these studies in breast cancer, the loss of RON in the TRAMP mouse model of prostate cancer led to a reduction in prostate tumor mass and tumor vascularization [25].

Tumor cell transplantation studies into RON-replete and RON-deficient (TK−/−) hosts were the first to describe a tumor cell-extrinsic role of RON in regulating tumor growth and metastasis [26]. In TK−/− hosts, tumor cell growth was significantly reduced compared to the growth of the same cells in wild-type hosts. Tumors in TK−/− hosts exhibited increased tumor cell apoptosis, macrophage infiltration, and altered cytokine production. Reciprocal bone marrow transplantation studies and transplantation into mice with a myeloid-specific loss of RON showed that myeloid RON lass was sufficient to block tumor growth, which could be restored by depletion of CD8+ T cells. These studies provided the first evidence that RON receptor expression in the tumor microenvironment (TME), specifically in macrophages, supports tumor growth in part via suppression of CD8+ T cells. Subsequent studies supported and expanded these findings, showing that RON loss in the host TME limited tumor cell metastases by mounting an anti-tumor CD8+ T cell response [27].

### 3.2. HGFL−/− Mouse Model

HGFL is known to play an important role in cell proliferation and differentiation and in regulating macrophage function. To assess the in vivo effects of HGFL, a GEMM was generated containing global HGFL loss (referred to as the HGFL−/− mice) [28]. HGFL−/− mice show no obvious phenotype abnormalities but do display a delay in macrophage activation. In assessing the role of HGFL in postnatal mammary gland development, HGFL loss resulted in smaller and fewer terminal end buds (TEBs), delayed postnatal mammary gland development, and significantly decreased macrophage recruitment to the TEBs by downregulating expression of chemoattractants such as Interleukin 6 (IL-6), monocyte chemoattractant protein-1 (MCP-1), and C-C chemokine receptor type 2 (CCR-2) [29]. STAT3 mRNA and phosphorylated STAT3 (pSTAT3) protein expression were significantly reduced in HGFL−/− mammary glands, suggesting that STAT3 activation may be the key pathway mediated by HGFL that influences macrophage recruitment and mammary gland development [29].

### 3.3. MMTV-RON Mouse Model

MMTV-RON transgenic mice are a GEMM [30] that was established to determine the functional consequences of RON overexpression in the mouse mammary epithelium. RON expression in this model is driven by the mouse mammary tumor virus (MMTV) promoter, followed by a RON cassette that contains the first three exons and introns of murine RON (mRON) genomic DNA followed by exons 4 through 19 of the RON cDNA. RON over-expression within the mouse mammary gland was found to be sufficient to induce highly metastatic mammary tumors with a 100% penetrance in female mice.

### 3.4. MMTV-RON^ΔMyeloid^ Mouse Model

To understand the role of myeloid RON in tumorigenesis, a myeloid deletion of RON in the MMTV-RON mouse model [18] was generated by crossing MMTV-RON RON^F/F^ mice with Lysozyme M-Cre mice [20,31,32]. Cre-mediated recombination resulted in the deletion of the endogenous RON gene in the myeloid cell lineage (monocytes, mature macrophages, and granulocytes). The conditional deletion of RON by Lysozyme M-Cre expressing mice was assessed at 83–98% deletion efficiency in macrophages and near 100% in granulocytes [31,32].

These crosses generated mice with a conditional deletion of RON in the myeloid compartment compounded with selective RON overexpression in the mammary epithelium (referred to as the MMTV-RON^ΔMyeloid^ mice). Compared to control MMTV-RON mice, MMTV-RON^ΔMyeloid^ mice with a myeloid deletion of RON had delayed mammary gland hyperplasia, delayed tumor initiation, slower tumor growth kinetics, and reduced lung metastases. Immunohistochemical analysis of mammary tumors from MMTV-RON^ΔMyeloid^ mice had reduced Ki67+ staining (cell proliferation) and increased Cleaved Caspase-3+ staining (apoptosis) compared to MMTV-RON RON^F/F^ control mice. Mammary tumors from MMTV-RON^ΔMyeloid^ mice, compared with controls, have increased macrophage recruitment based on F4/80+ staining, increased M1 macrophage numbers based on iNOS+ staining, and decreased M2 macrophages based on Arginase-1+ staining. Further, the mammary tumors from MMTV-RON^ΔMyeloid^ mice compared with controls show increased numbers of CD8a+ T cells and CD4+ T cells.

### 3.5. MMTV-RON^HGFL−/−^ Mouse Model

To study the role of HGFL in mice with RON-mediated mammary tumors, HGFL−/− mice were crossed to MMTV-RON mice to generate MMTV-RON^HGFL−/−^ mice [19]. In MMTV-RON^HGFL−/−^ mice, the onset of mammary hyperplasia and local tumor cell invasion was reduced compared to control mice. MMTV-RON^HGFL−/−^ mice also exhibited delayed mammary tumor initiation and metastasis, corresponding to decreased overall tumor burden compared to MMTV-RON mice. Further, HGFL loss significantly hindered RON kinase activity and downstream NF-κB and β-catenin signaling. In examining the TME, mammary tumors from MMTV-RON^HGFL−/−^ mice exhibited increased infiltration of F4/80 positive macrophages but also a significant decrease in Arginase 1 staining and a corresponding increase in iNOS staining. Additionally, increased recruitment, activation, and cytotoxic function of CD8+ T-cells was observed. These data highlighted the role of the endogenous HGFL in activating RON signaling to promote mammary tumorigenesis and metastasis with a corresponding alteration in the tumor microenvironment.

### 3.6. PyMT^HGFL−/−^ Mouse Model

The PyMT mouse model [33] involves the transgenic polyoma middle T antigen expression under the control of the MMTV promoter to target the polyoma oncoprotein selectively to the mammary glands of the mice. PyMT mice are a well-established spontaneous mouse model used to study breast cancer that mimics human breast cancers in that tumors develop in distinct stages and metastasize to the lungs [33]. In the PyMT mice, an upregulation of RON and HGFL expression was observed in the mammary glands of mice before 30 days of age. To understand the role of HGFL in supporting tumorigenesis in this mouse model, a global knockout of HGFL (PyMT^HGFL−/−^) [18,34] was generated by crossing the PyMT mice with HGFL−/− [28,29] mice. Mammary tumors from PyMT^HGFL−/−^ mice showed a significant reduction the tumor volume and lung metastasis compared to PyMT controls. Interestingly, losing HGFL in PyMT mice led to an increased recruitment of F4/80+ macrophages (increased iNOS + M1 macrophages and decreased Arginase-1 + M2 macrophages) and T cells [18].

### 3.7. PyMT TK^ΔEpithelial^ Mouse Model

To study the role of RON in driving breast cancer progression and metastasis, PyMT mice with a conditional loss of the RON tyrosine kinase (TK) domain selectively within the mammary epithelium were generated [35]. MMTV-Cre+/− [36] mice expressing Cre-recombinase in the mammary epithelium were crossed with RON^F/F^ mice to generate the MMTV-Cre+/− RON^F/F^ mice. To generate RON signaling loss within mammary epithelium cells in mammary-tumor-bearing mice, PyMT RON^F/F^ MMTV-Cre+/− (PyMT TK^ΔEpithelial^) mice were generated by crossing PyMT RON^F/F^ mice with MMTV-Cre+/− RON^F/F^ mice. Losing RON in mammary epithelial cells significantly reduced breast tumor growth and lung metastases compared to controls [35]. Further, immunohistochemical analysis indicated a significant increase in the number of apoptotic cells and decreased proliferation in mammary tumors within PyMT TK^ΔEpithelial^ compared to mammary tumors from PyMT RON^F/F^ controls. Upon accessing the immune microenvironment, macrophage marker F4/80 and M1 polarization marker iNOS were found to increase significantly in macrophage infiltration in PyMT TK^ΔEpithelial^ tumors compared to control mice. Further, tumors from PyMT TK^ΔEpithelial^ mice contained more cytotoxic CD8+ T cells compared to PyMT RON^F/F^ tumors. Overall, in the tumor microenvironment examined via immunohistochemistry and immunophenotyping, mammary tumors from PyMT TK^ΔEpithelial^ mice showed a significant increase in the percent of macrophages, NK cells, and CD8a positive cells present compared to PyMT RON^F/F^ tumors.

Taken together, in both PyMT and MMTV-RON models, RON signaling loss within the mammary epithelium or myeloid cell populations (see below) hinders breast cancer progression by overcoming an immunosuppressive tumor microenvironment to a more immune-enriched environment with increased M1 macrophage, NK cell, and cytotoxic CD8+ T cells or CD4+ T cells anti-tumor responses.

### 3.8. PyMT-RON^ΔMyeloid^ Mouse Model

The loss of Ron in myeloid cells was achieved by crossing mice containing a homozygous floxed tyrosine kinase domain of RON (RON^F/F^) to mice expressing Cre recombinase under the control of the lysozyme M promoter (RON^F/F^ LysCre) [37].

To examine myeloid RON loss in breast cancer development, the RON^F/F^ LysCre mice were crossed to PyMT mice to generate PyMT mice with a myeloid-specific RON loss (PyMT-RON^ΔMyeloid^). Mammary tumors from PyMT-RON^ΔMyeloid^ mice, compared to control mice, exhibited slower mammary tumor initiation and growth, less metastasis, fewer breast cancer stem cell (BCSC) numbers, and reduced BCSC self-renewal [20]. In terms of the impact on the immune populations in the tumor microenvironment, mammary tumors from PyMT-RON^ΔMyeloid^ mice had increased numbers of F4/80 and iNOS positive cells and decreased Arginase-1 positive cells in the tumor TME compared to mammary tumors from PyMT control mice. Interestingly, an increase in CD3+, CD8+, and CD4+ T cell populations and a significantly decreased B cell presence were also observed with myeloid RON loss in the PyMT model [20]. Overall, similar to the myeloid-specific RON loss in the MMTV-RON mouse model [18], conditional loss of RON in the myeloid compartment in PyMT mice resulted in impaired mammary tumorigenesis, impaired metastatic progression and enhanced recruitment of immune cell populations.

### 3.9. Hi-Myc Pb-RON Mouse Model

ARR_2_Pb-RON mice were generated using the prostate-specific probasin promoter with two androgen response elements from the probasin promoter arranged back-to-back to drive RON expression in the prostate [38]. RON overexpression under the ARR_2_Pb promoter led to the development of prostate adenocarcinoma and microinvasive mPIN lesions in mice [38]. Overexpression of RON in the prostate epithelium was further established in the Hi-Myc mouse model by crossing mice with RON overexpression in the prostate epithelium (ARR_2_Pb-RON) with Hi-Myc mice, a commonly used mouse model of human prostate cancer, to examine the effects of epithelial RON overexpression in a genetic model of prostate cancer [39]. Because the Myc gene copy number is typically elevated in human prostate cancer, the Hi-Myc mouse model uses the ARR_2_Pb promoter to drive Myc expression to recapitulate human prostate cancer in mice [40].

Prostates from Hi-Myc Pb-RON mice exhibited an increased tumor burden, incidence of prostate adenocarcinoma, and tumor cell proliferation [39] compared to prostates from control Hi-Myc mice. Regarding immune cell populations in Hi-Myc Pb-RON mice, prostate and subcutaneous tumors showed increased F4/80+ cells, Arginase-1 expression, and tumor cell CCL2 secretion compared to Hi-Myc controls [39]. Clodrosome-mediated depletion of macrophages and RON inhibition in combination with castration therapy in Hi-Myc Pb-RON mice resulted in reduced prostate tumor growth, significantly reduced numbers of F4/80+ cells in the TME, increased tumor cell death, and decreased tumor cell proliferation [39].

### 3.10. RON∆Epi/TRAMP Mouse Model

The transgenic adenocarcinoma of the mouse prostate (TRAMP) model has long been used to study prostate cancer development [41]. The TRAMP model was generated using the simian virus 40 (SV40) driven by the rat Probasin promoter in a C57BL/6 background [41,42]. Since SV40 expression is regulated by the Probasin promoter, the expression is restricted to prostate epithelial cells. As a result of this transgene, TRAMP+ mice develop prostatic intraepithelial (PIN) lesions after 8–10 weeks and subsequently develop adenocarcinoma by 22 weeks [41,42]. This model mimics more advanced stages of prostate cancer and includes metastatic prostate cancer progression [41].

As RON becomes overexpressed in prostate tumors of TRAMP+ mice compared to the prostates of wild-type mice, the TRAMP+ model is a useful tool to study the role of RON in prostate tumorigenesis [25]. Further, global RON loss in TRAMP+ mice mimicked phenotypes of global RON loss in breast cancer models [24,25].

To understand the role of RON selectively within the prostate epithelium, RON∆Epi mice were generated in the TRAMP+ background. To generate RON∆Epi/TRAMP+ mice, RON^F/F^ was crossed to Probasin Cre mice (PbCre) [23]. This yielded a prostate epithelial cell-specific knockout of RON (RON∆Epi mice). Similar to the RON−/− model, there was no distinct histological difference between the prostates of RON∆Epi and RON^F/F^ mice [43]. Next, RON^F/F^ mice were crossed with TRAMP+ mice, producing a TRAMP+ with loxP sites flanking the tyrosine kinase domain of RON (RON^F/F^/TRAMP+). Thus, crossing RON^F/F^/TRAMP+ mice with RON∆Epi mice generated a RON knockout specific to prostate epithelial cells of TRAMP+ mice (RON∆Epi/TRAMP+) [43].

RON∆Epi/TRAMP+ mice exhibited reduced prostate weight, metastases, PIN lesions, local invasion, micro blood vessel density, proliferation, and increased apoptosis compared to RON^F/F^/TRAMP+ controls. Furthermore, loss of epithelial RON expression promoted significant changes within the prostate TME. F4/80+ and iNOS + IHC staining was increased in the prostates of RON∆Epi/TRAMP+ mice compared to controls. mRNA expression of CXCL9, iNOS, and IL-12β, anti-tumor macrophage markers, was elevated, while mRNA expression of IFNγ, TNFα, ARG-1, TGFβ, IL-10, and VEGFa, pro-tumor macrophage markers, was significantly lower in TAMs isolated from the prostates of RON∆Epi/TRAMP+ compared to RON^F/F^/TRAMP+ mice. Additionally, TAMs isolated from RON∆Epi/TRAMP+ prostate tumors showed striking decreases in RON and Arginase-1 expression by Western blot compared to RON^F/F^/TRAMP control TAMs [43]. This decrease in RON expression on the TAMs of RON∆Epi/TRAMP+ mice suggests tumor-cell-intrinsic RON is important for presentation of the RON receptor on macrophages within the prostate TME.

Foundational studies from our laboratory have demonstrated RON signaling as a major mediator of breast and prostate tumorigenesis and progression through both tumor cell-intrinsic regulation and regulation of the TME. However, a significant knowledge gap exists in our understanding of the mechanisms by which RON mediates the crosstalk between tumor cells and immune cells or intra-immune cell populations. The tumor samples from those well-characterized murine models of cancer with deficiencies in RON expression in either tumor epithelial cells or in macrophages are invaluable to for further identification of novel RON-regulated pathways and genes which support an immunosuppressive TME.

## 4. RON Signaling in Macrophages

Canonically, RON signaling in macrophages is associated with wound healing. HGFL, also known as macrophage-stimulating protein (MSP), binds to and activates RON within macrophages, fibroblasts, and epithelial cells at wound sites to promote migration and close the wound [17,44,45]. Levels of HGFL are typically increased at wound sites, especially acute wounds [17,46]. RON is expressed in terminally differentiated macrophages but not in monocyte precursors. As such, much of macrophage RON signaling takes place in the native tissue resident microenvironment [11,16].

Macrophage polarization is an important indicator of the function and phenotype of macrophages. There are two primary classifications of activated macrophages: M1-polarized and M2-polarized macrophages. M1-polarized macrophages are considered more pro-inflammatory because these cells are intrinsically more cytotoxic and secrete cytokines that activate immune effectors to accomplish tumor-killing in the TME [47]. M2-polarized macrophages are considered more anti-inflammatory as these cells secrete cytokines that support the tumor and suppress the anti-tumor functions of other immune cells to limit tumor killing [47]. Expression of inducible nitric oxide synthase (iNOS) is commonly used as a marker of M1-polarization, while Arginase-1 is a common marker of M2-polarization. iNOS and Arginase-1 are mutually exclusive enzymes that compete for the same substrate within the urea pathway; arginine processed by iNOS yields nitric oxide and citrulline, whereas arginine processed by Arginase-1 generates L-ornithine and urea. Arginase-1 expression supports tumor growth through the production of polyamines, which are important for cell growth, and by out-competing iNOS to prevent the production of pro-inflammatory nitric oxide [48]. HGFL activation of RON signaling on macrophages has been shown to not only inhibit the expression of iNOS but also increase the expression of Arginase-1 to promote anti-inflammatory responses in surrounding tissues [3,16,49,50]. This connection between RON signaling and M2 polarization has been characterized in the contexts of prostate and breast cancer.

### 4.1. Prostate Cancer

Prostate cancer is the second leading cause of cancer-related death in men [51]. The predominant immune cell infiltrates within the prostate TME are macrophages [52,53]. Most of the macrophages within the prostate TME are M2 (pro-tumor) macrophages [52,53,54]. High M2 macrophage infiltration is associated with increased tumor staging, metastasis, and decreased survival for prostate cancer patients [52,54].

Similar to macrophages, increased RON expression within prostate tumors is associated with worse tumor staging and metastasis within human and mouse prostate tissue [39,43,55]. Macrophage infiltration into the prostate may be due in part to the ability of RON-expressing prostate cancer cells to secrete CCL2, a macrophage chemoattractant, into the prostate TME [39]. Additionally, RON-expressing prostate tumors are associated with more M2-polarized macrophages [39]. Paradoxically, loss of intratumoral RON expression within the TRAMP prostate cancer model also leads to enhanced macrophage infiltration, albeit with more M1 (anti-tumor) macrophages compared to TRAMP mice with intact RON signaling [43]. As a functional consequence of RON loss and subsequent loss of M2 macrophage polarization, prostate tumors in TRAMP mice with a conditional loss of RON in the prostate epithelium exhibit decreased adenocarcinoma, metastasis, and proliferation [43]. Using Hi-Myc derived RON overexpressing prostate cancer cells; our laboratory demonstrated that macrophage depletion, via clodronate treatment, significantly decreased prostate tumor growth similar to pharmacological RON inhibition [39]. However, the greatest tumor reduction came from combination treatments with clodronate and RON inhibition [39]. This highlights the importance of macrophages in prostate tumor development and suggests that combination treatments targeting macrophages and tumor cell-intrinsic RON signaling may be needed to combat prostate cancer growth.

To understand RON expression in the human prostate TME, RON expression was evaluated by IHC in prostate cancer tissue sections. RON expression was observed to be higher in the stroma of prostate adenocarcinoma samples compared to normal and benign tissues [26]. This is important because macrophages, particularly M2 macrophages, are known to remodel and promote pro-tumorigenic signaling within stromal tissues [26,56]. This increase in stromal RON expression was hypothesized to be due to the influx of M2 macrophages commonly seen in prostate cancer samples. To test myeloid RON function, mice with a conditional loss of RON in the myeloid compartment were utilized [26]. Loss of RON within macrophages resulted in decreased prostate tumor burden. Furthermore, prostate tumors grown in the presence of RON-deficient macrophages were more necrotic, indicating that RON expression in macrophages protects prostate tumors from death [26].

STAT3 is an important downstream signaling modulator of RON. Within macrophages, STAT3 signaling is important to support M2 macrophage polarization and subsequent immune evasion signaling within the TME [26]. RON-deficient macrophages have lower STAT3 phosphorylation and exhibit increased mRNA expression of M1 macrophage markers and T-cell regulatory genes associated with anti-tumor responses in the prostate TME [26]. Understanding the role of RON within the prostate TME and epithelial cells has the potential to provide additional therapeutic targets for patients suffering from this disease.

### 4.2. Breast Cancer

The RON receptor is overexpressed in about half of all human breast cancers and is an indicator of metastasis and worse overall survival [57]. The tumor microenvironment has been implicated in metastasis [58,59,60]. Expression of RON on breast tumor cells and macrophages presents an important axis to investigate the influence of RON signaling on breast cancer development and metastasis. Additionally, the fact that HGFL stimulation of RON on macrophages induces Arginase-1 expression and inhibits iNOS expression suggests a propensity for M2-polarization and, therefore, tumor-supportive functions of macrophages in the tumor microenvironment of breast cancers expressing high RON levels.

To examine the effect of RON signaling on breast tumor progression, RON was conditionally deleted in the mammary epithelium of PyMT mice (PyMT TK^ΔEpithelial^ mice). Mammary tumors from PyMT TK^ΔEpithelial^ versus PyMT control mice showed significantly less iNOS expression via immunohistochemical staining [35]. This indicates that macrophages in the mammary TME are more M1 polarized with mammary-specific RON loss, but the mechanism of this polarization is unknown.

Further, mammary tumors from PyMT TK^ΔEpithelial^ mice exhibited greater infiltration of macrophages, NK cells, and CD8 T cells into the TME than in RON-expressing PYMT tumors [35]. The increased iNOS and CD8 expression in the PyMT TK^ΔEpithelial^ tumors suggest greater inflammatory activation [35]. The available data support an anti-inflammatory/tumor-promoting bias associated with RON expression in the tumor proper. Additionally, CD8+ T cells harvested from the spleens of RON knockout mice significantly reduced experimental metastasis of breast cancer cells injected into the tail vein of immunodeficient mice [27]. Conversely, CD8+ T cells harvested from the spleens of wild-type mice had no effect on experimental metastasis. These studies indicate that RON signaling is responsible for suppressing CD8 T cell activation in the tumor microenvironment, although the mechanism associated with this is not clear.

RON loss in the mammary epithelium of PyMT TK^ΔEpithelial^ mice also resulted in delayed tumor progression, decreased tumor volume, and decreased metastases [35]. Importantly, RON loss in murine breast cancer cells implanted into syngeneic immune-competent mice did not engraft as well as isogenic RON-expressing cells. In contrast, however, both cell types engrafted equally well in immunocompromised mice, indicating the ability of the innate immune system to prevent implantation of breast cancer cells with RON loss [35]. This effect is posited to be due to RON signaling inhibiting the anti-tumor immune response extrinsically via tumor cell-intrinsic IRAK4 inhibition. RON directly interacts with IRAK4 to inhibit its pro-inflammatory effects in the TLR signaling pathway which suppresses type I interferon (IFN-I) signaling and thereby prevents activation of the anti-tumor response by innate immune cells [35]. RON-expressing cells were less susceptible to FasL and TRAIL killing by immune effectors than RON-deficient cells, suggesting that RON signaling in mammary epithelial cells confers resistance to immune cell attack [35]. These data suggest inhibition of inflammatory signaling within RON-expressing epithelial cells and extrinsically in the tumor microenvironment of RON-expressing breast tumors in PyMT mice.

R7 cells are a murine RON-expressing cell line isolated from a mammary tumor from MMTV-RON mice. R7 breast cancer cells cultured in conditioned media from bone marrow-derived macrophages (BMDMs) from syngeneic FVB mice with wildtype RON expression showed greater proliferation than R7 cells cultured in conditioned media from RON TK−/− BMDMs [20]. Additionally, macrophage RON loss in the PyMT-RON^ΔMyeloid^ mouse model showed decreases in mammary tumor cell proliferation, survival, cancer stem cell self-renewal, and metastasis [20]. Altogether, these data suggest that RON signaling within macrophages promotes the secretion of pro-tumorigenic factors that are not present when macrophages do not have RON expression. Further identification and functional characterization of these factors are areas for future study. Similarly, R7 cells co-cultured with RON-expressing BMDMs showed less apoptosis by Annexin V and Propidium Iodide analysis than R7 cells co-cultured with RON knockout BMDMs. Intriguingly, R7 cells co-cultured in conditioned media from WT RON or knockout RON BMDMs showed no difference in apoptosis, suggesting that the cell death seen previously was contact-dependent [20]. These data indicate that RON knockout in macrophages makes the macrophages more cytotoxic. However, whether the apoptosis evasion was due to signaling intrinsic to the RON overexpressing breast cancer cells or due to intrinsic inhibition of cytotoxicity within the RON-expressing macrophages bears further mechanistic study.

RON was knocked out in the myeloid population in PyMT-RON^ΔMyeloid^ mice to investigate the influence of macrophage RON-signaling as macrophages make up the predominant immune cell type in RON-expressing mammary tumors. RON-deficient macrophages in mammary tumors showed increased iNOS and decreased arginase-1 expression as compared to wild-type RON macrophages [20]. An increase in the M1-polarization marker with RON loss suggests that RON signaling promotes anti-inflammatory, pro-tumor signaling in macrophages. RON loss in myeloid cells, whether inducible or genetic, also resulted in greater CD8a T cell staining in the tumors as well as decreased B cell recruitment [20]. Overall, increased M1-polarization in breast tumors of genetic and inducible RON loss models, as well as increased CD8a T cell presence, suggests an anti-tumor microenvironment when RON is lost in the macrophages.

Global HGFL loss in the PyMT^HGFL−/−^ mouse model also led to an increase in CD8a T cells, F4/80+ and iNOS+ macrophages, and decreased Arginase-1 macrophages in mammary tumors [18]. Thus, the data from the PyMT TK^ΔEpithelial^, PyMT-RON^ΔMyeloid^, and PyMT HGFL−/− mouse models phenocopy one another, indicating the importance of this signaling axis in regulating the tumor microenvironment. The influence of RON-expressing macrophages on the tumor microenvironment in general remains to be characterized both phenotypically and mechanistically.

## 5. RON Signaling in Dendritic Cells

Dendritic cells (DCs) are critical members of the myeloid/monocyte lineage responsible primarily for initiating the adaptive immune response through their role as antigen-presenting cells (APCs). A recent paper has described the expression of RON on dendritic cells and explored the functional role of RON in this signaling context [61]. In treating Bone-Marrow-Derived Dendritic Cells (BMDCs) and splenic DCs with both LPS and a RON inhibitor (BMS777607), the authors observed the effect on MHCII and CD86, which serve as markers of DC maturation. Results of this study indicated that RON inhibition promoted March-1 transcription while also decreasing MHCII and CD86 expression, suggesting that RON signaling in DCs is important for LPS-induced DC maturation. Notably, this study utilized BMS777607, which, while targeting RON, also targets several other receptor tyrosine kinases, including Axl [62]. Axl is known to be expressed in monocyte populations, including both dendritic cells [63] and macrophages [64]. Considering that AXL has been implicated in the functionality of DCs using genetic models [63], this suggests that the effect shown in this paper may be more related to the activity of BMS777607 on AXL than on RON. Regardless, this paper shows that while RON expression in DCs is lower than in peritoneal macrophages, it remains expressed. The functional role of RON signaling both directly and indirectly on DCs remains unclear.

## 6. RON Signaling in Lymphoid Cells—T-Cells and NK Cells

While most work characterizing RON-mediated immune modulation involves discerning myeloid populations, particularly macrophages, the published literature has assessed RON-dependent modulations in lymphoid populations. Among these, natural killer (NK) cells are important populations that enable direct killing of tumor cells while also regulating the immune microenvironment. While previous work has shown that NK cells do not express RON, RON can indirectly regulate NK effector function, particularly the production of IFN-gamma through macrophage IL-12 secretion [65]. Additionally, work by our laboratory demonstrated that NK infiltration into mammary tumors was significantly increased with RON loss in the mammary epithelium of the spontaneous PyMT murine tumor model [35]. This same study also demonstrated that RON signaling in murine breast cancer cells enabled NK-mediated killing resistance in a co-culture model, further supporting the role of RON as an indirect regulator of NK effector function.

Following orthotopic transplantation of PyMT mammary tumor cells into mice with a global RON loss (TK^−/−^) or pharmacological inhibition of RON following transplantation, mammary tumor growth was inhibited compared to controls [13]. Interestingly, both RON loss or inhibition in the tumor cooperated with the T-cell checkpoint inhibitor CTLA-4 to reduce tumor growth with responses associated with increased intratumoral lymphocytes and higher T-cell activation markers. Combined RON and CTLA-4 targeting also lessened lung metastasis. In PyMT TK^ΔEpithelial^ mice, RON loss in the mammary epithelium resulted in increased infiltration of CD8+ T-cells similar to PyMT mice with a global RON loss (PyMT TK^−/−^) [35]. In a RON-driven murine mammary tumor model (MMTV-RON), loss of HGFL (MMTV-RON^HGFL−/−^) leads to a significant increase in the infiltration of CD8+ T-cells [19]. This finding was reinforced by HGFL loss in another murine mammary tumor model (PyMT) (PyMT^HGFL−/−^), which demonstrated increased infiltration of both CD8+ and CD4+ T-cells in mammary tumors [18]. This study also utilized a transplant model to demonstrate a similar phenotype of increased T-cell infiltration when HGFL loss was exclusively in the immune microenvironment. Notably, this study also employed RON loss selectively in the myeloid populations of MMTV-RON mammary tumor mice (MMTV-RON^ΔMyeloid^), which phenocopied the increased infiltration of CD8+ and CD4+ T-cells compared to control. Not only do these experiments demonstrate the immune exclusionary environment promoted by RON, but RON signaling in other immune populations can influence the adaptive T-cell response. Further research is needed to understand the influence of RON signaling, whether in macrophages or tumor cells, on T-cell activation, recruitment, and proliferation in the TME.

Interestingly, a recent study has also delineated the respective role of short-form RON (SF-RON) on the T-cell tumor populations. RON is known to have splice variants, including short-form RON (SF-RON), which lacks an extracellular domain due to an alternative start site in intron 10 [66]. In this work, orthotopic transplantation of PyMT tumor cells demonstrated that SF-RON loss increased TCF1+ CD4+ T-cells in metastatic lesions, which prevented outgrowth as well as an increase in the CD8+ T-cells which mediated the killing of metastatic cells [12]. The differences between SF-RON and full-length RON in regulating the tumor microenvironment is an emerging concept that warrants further inquiry.

Polarization of lymphoid populations, including T-cells and NK-cells, has become increasingly implicated in the mechanisms underlying tumorigenesis [67]. While T-cells and NK cells are classically considered tumor-suppressive in their direct tumor-killing and pro-inflammatory roles, immune polarization suggests distinct states in which these cells may promote tumor growth and development. While a number of in vivo studies have delineated tumor RON expression as a negative regulator of lymphoid infiltration, a considerable gap in knowledge remains regarding how RON may regulate the polarization states and effector function of these populations. As discussed previously, RON signaling perturbed both the IFN-gamma secretion and direct killing effects of NK cells [35,68]. While neither of these studies assessed the polarization state of these NK cells, the NK cells resemble a more pro-tumor type II NK cell, suggesting that RON signaling in the immune microenvironment may differentially regulate the NK polarization state. Along these lines, tumor growth factor beta (TGFβ) is known to promote the differentiation and recruitment of pro-tumor T-regulatory cells (T_reg_) [69]. While most phenotypic studies assessing RON-driven tumorigenesis have not directly characterized this immune population, knowing that RON expression in both epithelial and fibroblast populations promotes TGFβ production [70] suggests the role in RON promoting the polarization of T-lymphocytes toward an immunosuppressive T_reg_ state.

## 7. RON Signaling in Stroma/Fibroblasts

The stroma represents the connective tissues and cells supporting an organ and/or tumor. In homeostatic murine mammary tissue development, the expression of the RON ligand, HGFL, in both the mammary epithelium and surrounding stroma is important for ductal morphogenesis [29]. This study showed that HGFL is produced by both mammary epithelial and stromal compartments during development but particularly implicated the macrophage recruitment to the stroma surrounding terminal end buds (TEBs) in HGFL expressing mice compared to HGFL deficient (HGFL−/−) mice. This demonstrated that RON signaling in mammary development regulates the supportive microenvironment of the surrounding stroma. This epithelial–stromal interaction does not remain limited to homeostatic cellular processes but may also be implicated in RON-driven tumorigenesis. In breast cancer development, HGF/MET signaling undergoes distinct modulations in both the epithelial and stromal compartments during tumor initiation [71]. Considering that RON and HGFL share considerable homology and signaling pathways to MET/HGF supports the notion that they, too, may share this interaction. In line with this, a study of human pancreatic cancers showed increased phosphorylated-RON in the tumor cells compared to the surrounding stroma [72]. This finding delineates differential RON signaling dependent on the cellular compartment but supports the crosstalk between these compartments as being important for RON-driven tumorigenesis.

Research into RON-dependent mediators of stromal remodeling is an active research area. MMP12 was identified as directly regulated by RON in bladder cancer [73]. Using two human urinary bladder cancer cell lines with either RON short hairpin knockdown or RON overexpression, the authors demonstrated that MMP12 and HIF2-α were expressed downstream of RON activation of ERK/JNK signaling. Bladder cancer cells expressing a plasmid for RON showed increased migration and invasion compared to empty vector controls, yet siRNA inhibition of MMP2 in those cells containing RON plasmid significantly decreased these migratory and invasive phenotypes. These studies present important evidence that RON signaling promotes metastatic phenotypes in bladder tumor cells through stromal deconstruction, yet more characterization of this phenotype is necessary to understand the role of RON signaling in metastasis.

Fibroblasts represent a major cell population found in stromal compartments surrounding organs and tumors alike. RON is expressed on dermal fibroblasts and, when stimulated with its ligand HGFL, enhances wound healing processes mediated by fibroblast activity [44]. This study demonstrated that RON signaling in fibroblasts increased Collagen I/III production as well as MMP1-mRNA expression. Interestingly, fibroblast migration but not proliferation was increased with HGFL treatment. Furthermore, a study assessing the consequences of RON overexpression in interstitial fibroblasts (NRK49F) cells showed that transient transfection with a plasmid encoding RON increased the expression of fibrotic markers including TGFβ, αSMA, and fibronectin [70]. This study also employed siRNA silencing of Src, which decreased the expression of these same markers, thereby implicating Src downstream of RON as an important mediator in promoting kidney fibrosis. These studies support the role of intrinsic RON expression on fibroblast functionality and extracellular matrix remodeling, although additional studies are needed to further understand the crosstalk between pathogenic RON expression on fibroblast activity to promote tumorigenesis.

## 8. RON Signaling in Angiogenesis

Angiogenesis, the development of new blood vessels, is a critical process for homeostasis as well as the development of malignant tumors. These new vessels, resulting from angiogenesis, are essential for supplying nutrients to target tissues [74]. Moreover, the tumor microenvironment has been shown to strongly regulate this process, and itself may be influenced by vascularization in malignant contexts [75]. As RON expression in tumors serves a highly immunomodulatory role, it stands to reason that RON expression coordinately regulates angiogenic pathways. Accordingly, in both prostate and pancreatic cancer cells, RON expression is associated with increases in angiogenic chemokine production [55,76]. In prostate cancer cells, RON loss decreased microvessel density in an in vivo transplant model and RON expression was associated with angiogenic chemokines CXCL8, CXCL5, and CXCL1 [55]. Intriguingly, RON knockdown by siRNA in these prostate cancer cells did not alter VEGF levels. This contrasts with a study of RON on angiogenesis in pancreatic cancer in which HGFL treatment of RON-expressing cells significantly increased VEGF production [76]. Functionally, conditioned media from HGFL-treated RON-expressing pancreatic cancer cells promoted HMVEC microtubule formation. These studies are supported by RON modulations in the spontaneous PyMT murine mammary tumor model. RON loss (TK−/−) compared to wild-type PyMT mice demonstrated reductions in microvascular density [24]. Together, these studies show that chemokine production enabled by RON-expressing tumor cells is critical for promoting angiogenesis, which supports aggressive tumor development. Despite this knowledge, a gap remains in how RON-mediated immune signaling may also govern this important physiological process.

## 9. RON Modulated Interferon Signaling

RON activity in both homeostasis and cancer biology is associated with cytokine production and signaling in the immune microenvironment [11,77]. Of the critical immunological cytokines, interferons (IFN) are key players in both innate and adaptive immune responses, primarily in response to viral infection [78]. Likewise, cellular stress is another known inducer of IFN production and signaling [65,79,80]. There are three primary classes of IFNs, each of which signal through distinct receptor pairs which are differentially expressed dependent on the tissue and cell type [81], meaning that IFNs can have distinct effects dependent on the immunological context. Limited research has suggested that RON regulates the production of interferons in a context-dependent manner.

The type II interferon family includes IFN-gamma, which is conventionally produced by immune populations, including T-cells, NK cells, etc. IFN-gamma is also a critical cytokine in the polarization of macrophages to a more pro-inflammatory M1-like state. Research into the role of SF-RON using a model of acute liver injury induced by concanavalin A identified that SF-RON augments splenocyte IFN-gamma production [82]. This study delineated the respective roles of SF-RON compared to full-length RON, in which the loss of SF-RON alone in this model increased serum IFN-gamma levels compared to wild-type containing both full-length and SF-RON, but not other cytokines such as IL-4, IL-2 or IL-12. Notably, in this model, WT compared to full-length only RON expression showed no significant differences in macrophage or naïve T-cell distribution in splenocyte cell subpopulations. Loss of SF-RON alone resulted in increased hemorrhagic hepatocellular damage, dependent on IFN-gamma, suggesting that SF-RON-mediated IFN-gamma suppression in the liver is a critical stress-responsive pathway.

Another study also assessed the role of RON on IFN-gamma production in the context of innate immune signaling. Using LPS-challenged RON deficient mice (RON−/−), increased serum IFN-gamma production was observed compared to wild-type RON-expressing mice, largely due to altered NK cell IFN-gamma secretion [68]. This study demonstrated that septic shock susceptibility [49] in RON−/− mice was dependent on the IFN-gamma signaling. Furthermore, the authors demonstrated that treatment of macrophages with RON’s ligand, HGFL, reduced responsiveness to IFN-gamma through up-regulation of SOCS1 and SOCS3. RON-mediated promotion of these known negative regulators of IFN signaling may serve to limit the ability of IFN-gamma to polarize macrophages toward an M1 state. This is consistent with previous observations that RON signaling promotes and suppresses the pro-inflammatory M1 macrophage state and promotes a more anti-inflammatory M2 macrophage state [3,49,50].

The type I interferon family (IFN-I) is a broad class, including multiple variants of IFN-alpha and IFN-beta. IFN-I are often produced by epithelial cells in response to viral infection or cell stress [80]. Little research has been conducted on the role of RON in regulating IFN-I production and signaling, but a recent paper has identified the suppression of tumor intrinsic IFN-I production in RON-expressing breast cancer cells [35]. This inhibition is supported by the suppression of IRAK4 in RON-expressing cells, as well as interaction with the TLR/Myddosome complex. IRAK4 inhibition in RON low cells phenocopied the decreased IFN-I production of RON high cells. IRAK4 overexpression made RON high cells to Cd11b and NK cell killing in an ex vivo coculture. Together, this study suggests that RON-mediated suppression of IRAK4 enables IFN-I suppression while limiting the receptiveness to innate immune attack and potentiating metastatic potential. The fact that interferons can regulate cancer stem cell state [83], which is often associated with RON expression [34,84], seems to further support the notion that the suppression of tumor intrinsic interferon signaling is critical for potentiating aggressive breast cancer development.

Each of these studies, while distinct in their focus, demonstrates that RON regulates IFN production and signaling, often in a negative way. Strikingly, the mechanisms by which RON modulates IFN production and attenuates their activity seem to be highly context-dependent regarding the tissue and cell type of RON expression. Though studies remain limited, in both homeostasis and cancer contexts, the anti-inflammatory role of RON is partially mediated by suppression of IFNs, which warrants further evaluation.

## 10. Therapeutic Strategies and Challenges of Targeting RON for Cancer Treatment

Although RON ablation in either mammary epithelial or myeloid cell populations in vivo has been shown to increase the infiltration of various immune cell populations into the tumor microenvironment [14,16], key questions remain regarding the mechanisms by which RON promotes a pro-tumor microenvironment. Further studies are needed to elucidate the crosstalk between RON-expressing epithelial cells and macrophages, as well as to understand how different immune cell populations are recruited depending on the status of RON in myeloid or mammary epithelial cells. IFN-I play a central role in regulating both innate and adaptive immunity, and cancer cell-intrinsic IFN-I production can lead to differential responses to cancer therapy, depending on the strength and longevity of the responses. Notably, cancer cells that are resistant to cancer therapy often consistently produce IFN-Is to sustain chronic IFN-I responses [77]. Interestingly, RON has been shown to negatively regulate IFN-I production via suppression of IRAK4 [31]. However, the interplay between RON and IFN-I remains poorly understood, highlighting the need for further investigation into the mechanisms and consequences of RON-mediated IFN-I suppression.

Overexpression of RON has been observed in multiple tumor types. Moreover, the multifaceted role of RON in shaping the tumor microenvironment to support tumor growth and survival provides a strong rationale for pursuing RON-targeted therapies. Currently, multiple RON targeting inhibitors have been conducted in clinical trials. For instance, a phase II study (NCT01147484) [78] of Foretinib, a multi-kinase inhibitor of MET, RON, VEGFR2, and PDGFRβ—demonstrated preliminary evidence of activity and tolerability in metastatic, triple-negative breast cancer patients. Foretinib is also being investigated in combination with EGFR inhibitors (erlotinib or lapatinib) for various types of advanced cancers, including breast cancer [79,80]. Another agent, BMS-777607 [81], is a dual RON/MET inhibitor and showed a safety profile in a phase I trial (NCT01721148) for patients with advanced solid tumors [82]. Meanwhile, the RON-specific monoclonal antibody Narnatumab (IMC-RON8) was well tolerated in phase I trials (NCT01119456) but demonstrated limited antitumor activity at the tested dosing regimen [83].

Preclinical studies [85,86,87,88] indicate that pharmacological inhibition of RON, signaling via either kinase inhibitors or RON-specific monoclonal antibodies, can be therapeutically effective in vitro and in vivo. Nevertheless, important challenges persist. A deeper mechanistic understanding of how RON drives a pro-tumor microenvironment is needed, and combination approaches that target both the immune context and tumor cells may prove beneficial.

## 11. Conclusions

**Figure 1:**
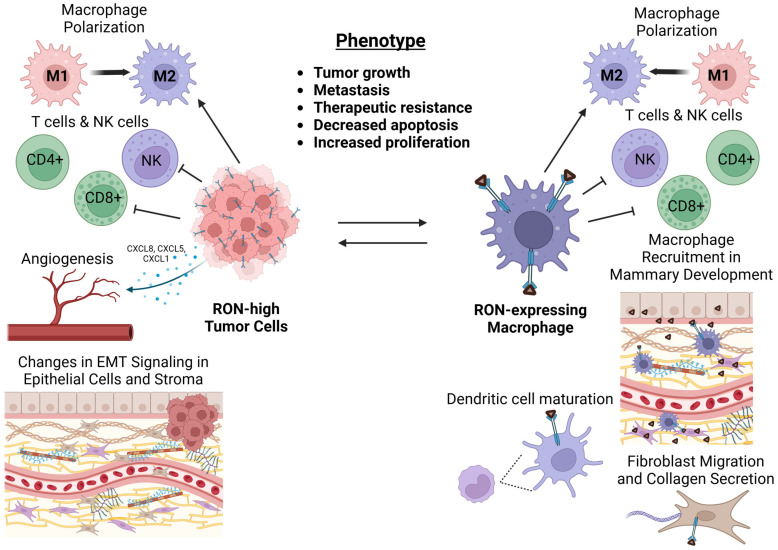
Crosstalk between RON-expressing tumor cells and macrophages enables a pro-tumor, immunosuppressive microenvironment. RON expression in cancer cells models the tumor microenvironment through regulation of immune populations as well as promotion of stromal remodeling and angiogenesis. RON expression in macrophages is critical in supporting tumor cell-intrinsic phenotypes and in supporting pro-tumor immune regulation. The crosstalk between tumor cells and macrophages enabled by RON promotes enhanced cancer cell growth, metastasis, and therapeutic resistance.

The RON receptor tyrosine kinase plays an important role in regulating the immune system in both homeostatic contexts and in malignancies such as cancer. While expressed at different levels across cell types, RON signaling is shown to have direct and/or indirect effects in a wide variety of immune populations. Correspondingly, these populations crosstalk with RON-expressing epithelial or tumor cells, which further augment immune function. In cancer progression, RON expression in epithelial cells and macrophages seems to be equally important for orchestrating the immunosuppressive tumor microenvironment that mediates the aggressive tumor phenotype driven by RON. Our laboratory has demonstrated that loss of RON in myeloid populations (PyMT-RON^ΔMyeloid^) nearly phenocopies RON loss in the mammary epithelium (PyMT TK^ΔEpithelial^) in PyMT mice (Table 1), resulting in a reduction in tumor growth and metastatic progression. Considering not only the role of macrophages in the innate immune system but also in regulating the function of the adaptive immune system, this population of immune cells is presented as a critical arm of RON-driven tumorigenesis. The crosstalk between RON-expressing tumor cells and macrophages appears to be the key axis governing the tumor microenvironment (Figure 1).

While most RON-related immune research has been in the context of macrophage activity, numerous gaps remain regarding the crosstalk between RON-expressing macrophages and other populations. Furthermore, many other immune populations have a rather limited characterization of both the direct and indirect influence of RON signaling. In discerning the pathways by which RON governs immune activation and function, enabling aggressive tumorigenesis, we can identify potential therapeutic avenues to target RON signaling. The RON receptor presents the opportunity to target both the tumor proper and the immune microenvironment, underscoring the importance of defining RON-dependent immune modulations if we are to make advances in treating RON-expressing malignancies.

## Figures and Tables

**Table 1:** RON-modulated genetically engineered mouse models demonstrate consistent tumor microenvironment phenotypes. Compared to the wild-type mouse control, “+” indicates an increase, and “−” indicates a decrease in the listed feature or phenotype corresponding to RON modulation. “No Change” indicates no difference between wild-type and the RON-modulated mouse model. “N/A” indicates that this feature or phenotype was not assessed in the corresponding mouse model.

Mouse Model		Phenotype		Immune Infiltration				Macrophage Polarization	
Oncogene	RON Modulation	Tumor Growth	Metastasis	Macrophage	CD4+ T-Cell	CD8+ T-Cell	NK-Cell	M1	M2
MMTV-RON	HGFL−/−	−	−	+	+	+	N/A	+	−
	RON^ΔMyeloid^	−	−	+	N/A	+	N/A	+	−
PyMT	HGFL−/−	−	−	+	+	+	N/A	+	−
	TK^ΔEpithelial^	−	−	+	No Change	+	+	+	−
	RON^ΔMyeloid^	−	−	+	+	+	N/A	+	−
	RON TK−/−	−	N/A	N/A	N/A	N/A	N/A	N/A	N/A
Hi-Myc	Pb-RON	+	N/A	+	N/A	N/A	N/A	N/A	+
TRAMP	RONΔEpi	−	−	+	N/A	N/A	N/A	+	−
	RON TK−/−	−	−	N/A	N/A	N/A	N/A	N/A	N/A
